# Phthalate Exposure and Health-Related Outcomes in Specific Types of Work Environment

**DOI:** 10.3390/ijerph110605628

**Published:** 2014-05-26

**Authors:** Branislav Kolena, Ida Petrovicova, Tomas Pilka, Zuzana Pucherova, Michal Munk, Bohumil Matula, Viera Vankova, Peter Petlus, Zita Jenisova, Zdenka Rozova, Sona Wimmerova, Tomas Trnovec

**Affiliations:** 1 Department of Zoology and Anthropology, Constantine the Philosopher University in Nitra, 949 74 Nitra, Slovakia; E-Mails: ipetrovicova@ukf.sk (I.P.); tomas.pilka@ukf.sk (T.P.); 2 Department of Ecology and Environmentalistics, Constantine the Philosopher University in Nitra, 949 74 Nitra, Slovakia; E-Mails: zpucherova@ukf.sk (Z.P.); vvankova@ukf.sk (V.V.); ppetlus@ukf.sk (P.P.); zrozova@ukf.sk (Z.R.); 3 Department of Informatics, Constantine the Philosopher University in Nitra, 949 74 Nitra, Slovakia; E-Mail: mmunk@ukf.sk; 4 Specialized Hospital of St. Zoerardus Zobor, 949 88 Nitra, Slovakia; E-Mail: matula@snzobor.sk; 5 Department of Chemistry, Constantine the Philosopher University in Nitra, 949 74 Nitra, Slovakia; E-Mail: zjenisova@ukf.sk; 6 Institute of Biophysics, Informatics and Biostatistics, Slovak Medical University, 833 03 Bratislava, Slovakia; E-Mail: sona.wimmerova@szu.sk; 7 Department of Environmental Medicine, Slovak Medical University, 833 03 Bratislava, Slovakia; E-Mail: tomas.trnovec@szu.sk

**Keywords:** urinary phthalate metabolites, occupational exposure, pulmonary functions, anthropometry

## Abstract

Many toxic substances in the workplace can modify human health and quality of life and there is still insufficient data on respiratory outcomes in adults exposed to phthalates. The aim of this work was to assess in waste management workers from the Nitra region of Slovakia (*n* = 30) the extent of exposure to phthalates and health-related outcomes. Four urinary phthalate metabolites mono(2-ethylhexyl) phthalate (MEHP), monobutyl phthalate (MnBP), monoethyl phthalate (MEP) and monoisononyl phthalate (MiNP) were determined by high-performance liquid chromatography with mass spectrometry (HPLC-MS/MS). Urinary concentration of MEHP was positively associated with ratio of forced expiratory volume in 1 s to forced vital capacity % (FEV_1_/FVC) (*r* = 0.431; *p* = 0.018) and MiNP with fat free mass index (FFMI) (*r* = 0.439; *p* = 0.015). The strongest predictor of pulmonary function was the pack/year index as smoking history that predicted a decrease of pulmonary parameters, the FEV_1_/FVC, % of predicted values of peak expiratory flow (PEF % of PV) and FEV_1_ % of PV. Unexpectedly, urinary MEHP and MINP were positively associated with pulmonary function expressed as PEF % of PV and FEV_1_/FVC. We hypothesize that occupational exposure to phthalates estimated from urinary metabolites (MEHP, MiNP) can modify pulmonary function on top of lifestyle factors.

## 1. Introduction

Some work environment factors can greatly affect human health. Negative effects are especially pronounced when workers are exposed to multifactorial conditions and noxious agents [1]. Phthalates —the esters of 1,2-benzenedicarboxylic acid, constitute a group of man-made chemicals having many industrial applications. As plasticizers, phthalates are additives which improve the flexibility, processability and softness of vinyl [2]. Phthalates are not bonded with covalent bonds to the polymer chains, but rather their molecules are embedded between the polymer chain molecules, so they can leach out or evaporate into air or become part of dust and airborne particles. Due to this fact, they have become one of the major indoor pollutants [3,4,5] and play a role as important environmental factors in the pathogenesis of negative health outcomes. Other possible routes of exposure are through contaminated foodstuffs, mainly through packaging materials, or dermal contact with cosmetics containing phthalates [6,7,8].

Phthalate exposure was found to be related to various adjuvant or inflammatory responses of inflammatory cells [9] and it was shown that phthalates may contribute to airway remodelling [10] or may affect respiratory health [4,11,12,13,14]. Some studies show that employment in the polyvinyl chloride (PVC) fabrication industry may be associated with both obstructive and restrictive ventilatory effects [15] and pointed to a potential toxicological effect of vinyl and PVC [16,17]. Exposure to the thermal degradation products of PVC and phthalic acid esters leads to symptoms affecting the eyes and upper airways, probably associated with the thermal degradation products of PVC. However no significant differences were found between exposed and control subjects with regard to spirometry [18].

Di(2-ethylhexyl) phthalate (DEHP) has been associated in epidemiological studies with the development of wheezing and allergic airway diseases [10,19,20]. At high concentrations, mono (2-ethylhexyl) phthalate (MEHP) can have acute airway irritant effects in mice [21]. In polyvinylchloride fabrication workers decreases in adjusted cross-shift ratio of forced expiratory volume in 1 s to forced vital capacity (FEV_1_/FVC), pre shift FEV_1_/FVC, and prevalence of chronic cough and chronic phlegm were observed [19]. The complete role of phthalates in respiratory diseases still remains to be established [14], but indoors emissions from plastic `materials may have adverse effects on the lower respiratory tract [17]. Increasing but limited evidence for the effects of phthalate exposure on respiratory health has demanded attention for further investigation [11,14,22]. Because of the presence of phthalates in a great number of materials and products incommon use, the opportunity of exposure in everyday life is high. This risk is considerably higher in waste management workers because they are exposed to a wide range of different plastic materials in mixtures of substances which can influence their leaching or evaporation. The aim of this work was to study the potential effect of exposure to phthalates and health-related outcomes in waste management workers.

## 2. Methods

### 2.1. Study Population

The cohort consisted of full time community services workers (20 men and 10 women) working in the Nitra region (Slovakia). Men were employed on average 7.9 years and women 5.6 years. Subjects with obstructive airway disease (OAD) and incomplete answers to the questionnaire were excluded. Men worked as waste truck drivers and co-drivers, experiencing both the waste-loading exposure and the air pollution emissions from road transport (nitrogen oxides, carbon monoxide, carbon dioxide, sulphur dioxide, persistent organic pollutants and phthalates). Women experienced exposure, mostly to phthalates, polychloroethene, and bisphenol A, in the course of sorting and processing waste substances for recycling. Participation was voluntary and there was a possibility to withdraw from participation at any time during the study. The study protocol was approved by the institutional review board of the Slovak Medical University. All human participants gave written informed consent prior to the study, and agreed to provide samples of urine during the shift, complete questionnaires and allow the researchers to take measurements and also to process their medical and personal records and data.

### 2.2. Phthalates Analyses

We collected urine samples (2 × 2 mL) from all volunteers during a work shift break (*i.e.*, workers began work no earlier than 6:00 am and worked at least 8 h per shift) and stored them in a transport box at 2–6 °C and in the laboratory in a deep freezer at −73 °C until analysis. Urinary levels of mono(2-ethylhexyl) phthalate (MEHP), mono-*n*-butyl phthalate (MnBP), monoisononyl phthalate (MiNP), and monoethyl phthalate (MEP) as metabolites of the parent phthalates di(2-ethylhexyl) phthalate (DEHP), di-*n-*butyl phthalate (DnBP), diethyl phthalate (DEP) and di-*iso*-nonyl phthalate (DiNP) were measured. We used high performance liquid chromatography (HPLC) and tandem mass spectrometry (MS/MS) (Infinity 1260 and 6410 triple quad instrumentas, Agilent, Santa Clara, CA, USA) using a modification of the method reported by Silva [23]. Analytical standards of MEHP, MnBP, MiNP and MEP (>99%), their isotopically labelled internal standards (ring-1,2-^13^C_2_, dicarboxyl-^13^C_2_, 99%) were purchased from Cambridge Isotope Laboratories, Inc. (Andover, MA, USA). Briefly, 1 mL of urine was thawed, buffered with ammonium acetate, spiked with isotope labelled phthalate standards, β-glucuronidase enzyme (Roche, Mannheim, Germany) and incubated (37 °C). After deconjugation samples were diluted with phosphate buffer (NaH_2_PO_4_ in H_3_PO_4_) and loaded onto SPE cartridges (ABS Elut Nexus, Agilent). Cartridges were conditioned with acetonitrile followed by phosphate buffer before extraction. To remove hydrophilic compoundd SPE cartridges were flushed with formic acid and HPLC grade water. Elution of analytes was performed using acetonitrile and ethylacetate. Eluate was dried by nitrogen gas and reconstituted with 200 µL of H_2_O. For HPLC was used Agilent Infinity 1260 liquid chromatograph equipped with ZORBAX Eclipse plus phenyl-hexyl column. Separation was done using non-linear gradient program. Agilent 6410 triplequad with electro-spray ionization was used for mass specific detection of phthalate metabolites. Instrumental settings were as follows: spray ion voltage (−3800 V), nitrogen nebulizer gas pressure (8 psi), and nitrogen curtain gas pressure (7 psi), capillary temperature (430 °C), and collision gas (nitrogen) pressure (1.5 mTorr). Precursor and product ions, collision energies, retention times and limits of detection (LOD) are shown in Table 1.

**Table 1:** Phthalate monoesters: Chromatographic and mass spectrometric parameters.

Compound Name	Precursor Ion	Product Ion	Fragmentor (V)	Collision Energy (V)	RT (min)	LOD (ng/mL)
MiNP	291.2	141.2	95	13	15.2	8.12
MiNP-labelled	295.3	79	95	13	15.2	
MEHP	277.1	133.9	90	14	14.7	0.81
MEHP-labelled	281.1	137.1	90	14	14.7	
MEP	193.0	77.1	60	15	6.2	5.02
MEP-labelled	197.1	79.0	60	15	6.2	
MnBP	221.1	76.9	90	10	11.8	3.23
MnBP-labelled	225.1	78.8	90	10	11.8	

**Note:** MiNP, monoisononyl phthalate; MEHP, mono (2-ethylhexyl) phthalate; MEP, monoethyl phthalate; MnBP, mono-n-butyl phthalate; RT, retention time; LOD, limit of detection.

### 2.3. Anthropometry

We collected anthropometric measures using standard methods: body height (by A 319 TRYSTOM, Ltd., Olomouc, Czech Republic), waist girth and hip girth (by a flexible non-elastic measuring tape). Body-mass index (BMI), waist-to-height ratio (WHtR), waist to hip ratio (WHR), fat mass index (FMI) and fat free mass index (FFMI) was calculated. Body weight, body fat percentage, muscle mass percentage, and visceral fat level were estimated by Omron BF510 (Kyoto, Japan) by bio-electrical impedance analysis, using a 50 kHz current source with electrodes on each hand and foot.

### 2.4. Spirometry

Spirometric testing was done by a trained technician according to 2005 European Respiratory Society/American Thoracic Society recommendations [24] using a Spirolab II (MIR, Rome, Italy) spirometer and Winspiro PRO software. We recorded the best result from three consecutive pulmonary function tests. Values for forced expiratory volume in 1s (FEV_1_, L), forced vital capacity (FVC, L), a ratio of FEV_1_ to FVC (FEV_1_/FVC, %) and peak expiratory flow (PEF) were obtained. In addition we calculated FEV_1_, FVC and PEF parameter reproducibility and diagnosed chronic obstruction pulmonary disease (COPD) according to GOLD definition [25].

### 2.5. Statistics

An association between phthalate exposure and pulmonary function was examined by correlation analysis. Backward multiple linear regression analysis to determine the association between urinary phthalate metabolites and pulmonary function (FEV_1_/FVC, % PV of PEF, % PV of FEV_1_ and % PV of FVC) were used. We considered the predictor to be significant when *p* value was ≤0.05. We used statistics program SPSS 16 (Softonic International S.L., Chicago, IL, USA).

## 3. Results

The characteristics of subjects are described in Table 2. All participants (*n* = 30) were in permanent, full-time position in communal services in the Nitra region, Slovakia. Men had been employed on average 94.8 months (*n* = 20) and women 66.6 months (*n* = 10). The percentage of smokers and ex-smokers in the group was similar, 55% and 60%, respectively. Pack/year index (p/y) was much higher in men (21.95 ± 18.67) than in women (7.13 ± 8.31).

Spirometry results suggest a decrease of pulmonary functions (especially FEV_1_ and FEV_1_/FVC) associated with respiratory disease. We detected mild to severe COPD symptoms in 20% (*n* = 6; p/y = 24.08 ± 21.88) and symptoms of chronic bronchitis (CHB) in 50% (*n* = 15; p/y = 12.80 ± 10.96) of subjects.

Table 3 summarizes data on concentration of phthalate monoesters in urine of the 30 waste management workers collected during a day shift. We found that the concentration of the metabolite MEHP is the highest, followed by the metabolites MnBP, MEP and MiNP.

We observed several relationships between anthropometric and respiratory parameters not related to phthalate exposure. Thus, we observed a significant decrease of FEV_1_/FVC associated to pack/year index (*r* = −0.425; *p* = 0.019) and sagittal chest diameter (*r* = −0.410; *p* = 0.025). The results of our study suggest significant associations between decreases in FVC % of predicted value with transverse chest diameter (*r* = −0.406; *p* = 0.026) and BMI (*r* = −0.385; *p* = 0.036). We only observed associations between decreases in FEV_1_ % of predicted values with transverse chest diameter (*r* = −0.439; *p* = 0.015) and BMI (*r* = −0.375; *p* = 0.041).

We observed an association between FFMI and increased PEF % of PV (r = 0.362; *p* = 0.049). In agreement with the purpose of the study, we observed that the urinary concentration of MEHP was positively associated with FEV_1_/FVC (*r* = 0.431; *p* = 0.018). Values of WHR were inversely associated with urinary levels of MEHP (*r* = −0.362; *p* = 0.049). We also found correlations between WHtR (*r* = −0.357; *p* = 0.057) and waist circumference (*r* = −0.347; *p* = 0.060) and decreasing concentration of MEHP. We observed association between FFMI and increase of MiNP concentration (*r* = 0.439; *p* = 0.015).

**Table 2:** Characteristics of the study group cohort from waste management workers in Nitra.

Parameter	Men (*n* = 20)		Women (*n* = 10)	
Urban	*n* = 13		*n* = 8	
Rural	*n* = 7		*n* = 2	
	Average	SD	Average	SD
Age	46.0	8.0	45.6	11.0
Body height	178.3	5.95	162.4	10.49
Weight	95.2	14.37	70.0	8.51
BMI	29.9	4.3	26.7	3.96
Waist circumference	106.04	11.4	88.6	13.88
Hip circumference	107.78	6.87	104.2	4.8
WHR	0.99	0.07	0.82	0.1
WHTR	0.6	0.1	0.5	0.1
Body fat percentage	29.6	6.30	38.7	9.16
Muscle mass percentage	32.2	3.18	25.89	4.84
Visceral fat level	13.9	4.8	7.5	3.0
FMI	9.1	2.9	10.7	3.7
FFMI	20.8	1.6	16.1	0.95
FVC	5.0	0.97	3.4	1.12
FVC % of PV	105.19	16.46	109.2	23.7
FEV_1_	3.73	0.91	2.6	0.68
FEV_1_ % of PV	96.57	20.46	97.5	17.71
FEV_1_/FVC	74.1	8.39	77.8	10.32
MVV	81.9	23.97	46.7	18.32
MVV % of PV	60.32	16.71	100.6	12.99
VC	4.37	0.82	3.2	1.01
VC % PV	88.34	15.04	101.6	21.22
PEF	8.03	2.08	4.81	0.62
PEF % of PV	87.63	21.01	74.99	9.68

Notes: BMI, Body-mass index; WHR, waist to hip ratio; WHtR, waist-to-height ratio; FMI, fat mass index; FFMI, fat free mass index; FVC, forced vital capacity (L); FVC % of PV, % of predicted values of forced vital capacity; FEV_1_, forced expiratory volume in 1s (L); FEV_1_ % of PV, % of predicted values of forced expiratory volume in 1s; FEV_1_/FVC, ratio of forced expiratory volume in 1 s to forced vital capacity (%); MVV, maximum voluntary ventilation (L); MVV % of PV, % of predicted values of maximum voluntary ventilation; VC, vital capacity (L); VC % of PV, % of predicted values of vital capacity; PEF, peak expiratory flow (L); PEF % of PV, % of predicted values of peak expiratory flow; n, frequency; p/y, pack/years index.

**Table 3:** Concentration of phthalate metabolite (ng/mL) in urine of 30 waste management employees.

Phthalate Metabolite	*n*	Mean ± SD	LOD	*n* < LOD (%)	Percentiles				
					25th	50th	75th	90th	95th
MEP	30	68.32 ± 43.74	5.02	23.33	40.84	49.20	77.26	89.60	344.45
MnBP	30	71.42 ± 90.19	3.23	16.67	39.55	67.13	92.84	128.65	130.04
MEHP	30	15.37 ± 20.09	0.81	13.33	2.72	5.94	17.43	50.60	60.71
MiNP	30	1.47 ± 4.47	8.12	90.00	<LOD	<LOD	<LOD	12.77	14.89

Notes: MEP, monoethyl phthalate; MnBP, mono-n-butyl phthalate; MEHP, mono (2-ethylhexyl) phthalate; MiNP, monoisononyl phthalate; LOD, limit of detection, n, frequency.

Table 4 summarizes the results of backward multiple linear regression analysis used for identification of factors associated with pulmonary function. We are presenting only regressions with significant results. The strongest predictor of pulmonary function was the p/y index. Smoking history predicted a decrease of the following pulmonary parameters: index FEV_1_/FVC, FEV_1_ % of PV and PEF % of PV. Unexpectedly, the concentration of two urinary phthalate metabolites, MEHP and MiNP, were positively associated with pulmonary function (in PEF % of PV and FEV_1_/FVC). Anthropometric parameters FFMI, BMI and transversal chest diameter were associated with pulmonary function. BMI was inversely associated with pulmonary parameters in FEV_1_ % of PV and in FVC % of PV while FFMI was positively associated with in PEF % of PV.

**Table 4:** Results of backward multiple linear regression analysis.

Pulmonary function	Parameter	B	Std. Error	Beta	t	Sig
PEF % of PV ^a,b^	MiNP ng/mL	1.854	1.022	0.305	1.815	0.081
	p/y	−0.429	0.202	−0.357	−2.125	0.043
	p/y	−0.474	0.195	−0.395	−2.429	0.022
	FFMI	0.718	0.332	0.352	2.164	0.040
FEV_1_/FVC ^a,c^	MEHP ng/mL	0.163	0.069	0.366	2.358	0.026
	p/y	−0.250	0.090	−0.433	−2.791	0.010
FEV_1_ % of PV ^a,d,e^	p/y	−0.612	0.201	−0.499	−3.046	0.005
FVC % of PV ^d,e^	BMI	−1.641	0.744	−0.385	−2.207	0.036
	transverse chest diameter	−2.274	0.969	−0.406	−2.348	0.026

Notes: The dependent variables were the parameters of pulmonary function (PEF % of PV, % of predicted values of peak expiratory flow; FEV_1_/FVC; ratio of forced expiratory volume in 1 s to forced vital capacity; FEV_1_ % of PV, % of predicted values of forced expiratory volume in 1 s; FVC % of PV, % of predicted values of forced vital capacity) and the independent variables concentration of urinary phthalate (MEHP, monoethylhexyl phthalate; MiNP, monoisononyl phthalate; MnBP, mono-n-butyl phthalate; MEP, monoethyl phthalate), smoking history (p/y, pack/year index) and various anthropometric characteristic (FFMI, fat free mass index; transverse chest diameter; saggital chest diameter; BMI, body mass index). ^a^ MEP (ng/mL, MnBP (ng/mL), MEHP(ng/mL), MiNP (ng/mL), pack/year index; ^b^ MEP (ng/mL, MnBP (ng/mL), MEHP(ng/mL), MiNP (ng/mL), pack/year index, FFMI; ^c^ MEP (ng/mL), MnBP (ng/mL), MEHP (ng/mL), MiNP (ng/mL), pack/year index, saggital chest diameter; ^d^ MEP (ng/mL, MnBP (ng/mL), MEHP (ng/mL), MiNP (ng/mL), pack/year index, BMI; ^e^ MEP (ng/mL), MnBP (ng/mL), MEHP (ng/mL), MiNP (ng/ml), pack/year index, transverse chest diameter. B is regression coefficient and the negative sign of regression coefficient means that the variables are negatively associated.

## 4. Discussion

Many toxic substances encountered in the workplace can modify human health and quality of life. We have shown herein that there is still insufficient data on respiratory outcomes for adults exposed to phthalates. We detected that mean concentrations for urinary MEP, MnBP and MEHP are positively skewed, which means that in community services workers high levels prevail in the upper quantiles.

Urinary MEHP concentrations in our study (median 5.94 ng/mL) are comparable with workers in a factory with polymer moulding (pre-shift urine sample) and workers in DEHP manufacturing (post-shift urine sample). However they are lower than in other factories included [26]. In comparison with the environmentally exposed general population [2,27,28] we detected a higher concentration of MEHP in urine (median 5.94 ng/mL; 95th percentile 60.71 ng/mL). In other studies on the general population the median was higher but lower 95 percentile values of MEHP concentration (10.3 ng/mL; 37.9 ng/mL [29] and 7.6 ng/mL; 33.6 ng/mL [30]) were observed. However, the values from European countries remain higher than those reported by U.S. authors, which can be explained by substitution of DEHP by other phthalates (DiNP/DiDP) in the United States [30].

The concentration of MiNP was low and close to the detection limit, with detection frequencies of 10% (median < LOD; 95th percentile 14.89 ng/mL). It is comparable to results of the Canadian Health Measures Survey [31], but higher than concentration values in a Danish mother-child pairs study [32]. Exposure estimates based on the concentrations of monoester metabolites of DiNP and DiDP probably underestimates human exposure [33]. It also should be noted that MiNP is not the major metabolite of the parent phthalate as it is converted into several other secondary metabolites prior to excretion [34], so low MiNP detection frequencies do not necessarily suggest low population exposure level to DiNP.

The concentration of MnBP in our study (median 67.13 ng/mL; 95th percentile 130.04 ng/mL) is comparable with an analysis in adult German population [27], but higher than results realised on the general German population [28].

In contrast with the results of National Health and Nutrition Examination Survey (NHANES) [2,23] we recorded lower median and 95 percentile concentration values of MEP (49.20 ng/mL; 344.45 ng/mL), but comparable with investigations on the German general population [28]. Based on the questionnaire and the low socioeconomic status of waste management workers, lower urinary concentrations of MEP were not surprising, because MEP is commonly used in cosmetics, personal care and sun screen products.

Based on correlation analyses, in our study we found an association between phthalate exposure and respiratory health, in particular between MEHP and MiNP and PEF % of PV and FEV_1_/FVC. This is not in agreement with data from Third National Health and Nutrition Examination Survey (NHANES III) [14], where no correlation was detected between MEHP and FEV_1_/FVC or effects of MEHP on the respiratory tract. On the other hand, an association between urinary concentrations of the metabolites of DEP and of DnBP to decreased lung function was reported [14]. Based on associations found between PVC and bronchial obstruction [10] we assume that the presence of COPD and chronic bronchitis symptoms in our cohort could be associated to phthalate exposure, however it is crucial to take the effect of smoking into account. We speculated on possible modulatory role of phthalates as endocrine disrupting chemicals, which could affect body composition (especially lipid accumulation and fat distribution) and subsequently pulmonary functions. This speculation has yet to be examined in larger samples.

Our results agree with the Korean Elderly Environmental Panel (KEEP) study [35], in which no significant association was found between MnBP and any of the pulmonary function measurements. Urinary levels of MEHP and MiNP were positively associated with PEF % of PV and FEV_1_/FVC, which is in contrast to the KEEP survey, where an inverse association between DEHP and FEV_1_/FVC was detected. Nevertheless some differences in the analytical approach in the KEEP survey compared to us should be noted. They considered DEHP as the sum of the secondary metabolites mono-(2-ethyl-5-hydroxyhexyl) phthalate and mono-(2-ethyl-5-oxohexyl) phthalate, whereas we only evaluated the primary metabolite MEHP.

There is growing interest in the possibility that endocrine disrupting chemicals, such as phthalates, may affect obesity-related pathways [36]. Indeed, association of concentrations of several prevalent phthalate metabolites with abdominal obesity and insulin resistance was found in a cross-sectional study of US men [37]. In the National Health and Nutrition Examination Survey (NHANES 1999–2002) [38] MEHP was inversely related to BMI, while in our study BMI was inversely associated with WC, WHR and WHtR. We also found an association between increased MiNP concentrations and FFMI. This association would suggest that phthalates may contribute to the body composition.

Our study has several important limitations. The small number of participants (*n* = 30) could distort results. Another weakness of our study is also the great number of samples with concentration <LOD for MiNP which limited the statistical significance of our data. Further single spot-urine measurement of phthalates does not necessarily reflect the whole exposure history of each participant. Exposure sources may vary over time based on dietary intake, use of personal care products and other factors. A final limitation of study is that we measured only simple monoesters of DEHP and DiNP in urine however oxidative metabolites are much more sensitive biomarkers of phthalate exposure. In the future, special attention has to be paid to the analyses of the oxidative metabolites of long-chain phthalates (DEHP, DiNP) and new potential metabolites (DiNP) [39], which are more suitable to capture the average background exposure. The availability of a greater number of participants would have improved our results.

## 5. Conclusions

We have shown in a cross sectional study that waste management workers are exposed to a range of phthalates. Our results suggest that occupational exposure to phthalates as endocrine disrupting compounds, evaluated on the basis of urinary metabolites (MEHP and MiNP) on top of lifestyle factors, can modify pulmonary function (PEF % of PV and FEV_1_/FVC) and body composition (FFMI, but not BMI).

## References

[1] Factors of Work Environment and Health Status of Workers in Condition of Plastic Industry of Slovak Republic. http://ehp.niehs.nih.gov/ehbasel13/p-3-16-17/.

[2] Blount B.C., Silva M.J., Caudill S.P., Needham L.L., Pirkle J.L., Sampson E.J., Lucier G.W., Jackson R.J., Brock J.W. (2000). Levels of seven urinary phthalate metabolites in a human reference population. Environ. Health Perspect..

[3] Bornehag C.G., Sundell J., Weschler C.J., Sigsgaard T., Lundgren B., Hasselgren M., Hagerhed-Engman L. (2004). The association between asthma and allergic symptoms in children and phthalates in house dust: A nested case–control study. Environ. Health Perspect..

[4] Jaakkola J.J., Knight T.L. (2008). The role of exposure to phthalates from polyvinyl chloride products in the development of asthma and allergies: A systematic review and meta-analysis. Environ. Health Perspect..

[5] Kolarik B., Naydenov K., Larsson M., Bornehag C.G., Sundell J. (2008). The association between phthalates in dust and allergic diseases among Bulgarian children. Environ. Health Perspect..

[6] Heudorf U., Mersch-Sundermann V., Angerer J. (2007). Phthalates: Toxicology and exposure. Int. J. Hyg. Environ. Health.

[7] Stanley M.K., Robillard K.A., Staples C.A. (2003). Anthropogenic compounds. Part 3Q. Phthalate Esters. The Handbook of Environmental Chemistry.

[8] Koniecki D., Wang R., Moody R.P., Zhu J. (2011). Phthalates in cosmetic and personal care products: Concentrations and possible dermal exposure. Environ. Res..

[9] Tsai M.J., Kuo P.L., Ko Y.C. (2012). The association between phthalate exposure and asthma. Kaohsiung J. Med. Sci..

[10] Jaakkola J.J., Oie L., Nafstad P., Botten G., Samuelsen S.O., Magnus P. (1999). Interior surface materials in the home and the development of bronchial obstruction in young children in Oslo, Norway. Am. J. Public Health.

[11] Jaakkola J.J., Verkasalo P.K., Jaakkola N. (2000). Plastic wall materials in the home and respiratory health in young children. Am. J. Public Health.

[12] Oie L., Nafstad P., Botten G., Magnus P., Jaakkola J.J.K. (1999). Ventilation in homes and bronchial obstruction in young children. Epidemiology.

[13] Ponsonby A.L., Dwyer T., Kemp A., Cochrane J., Couper D., Carmichael A. (2003). Synthetic bedding and wheeze in childhood. Epidemiology.

[14] Hoppin J.A., Ulmer R., London S.J. (2004). Phthalate exposure and pulmonary function. Environ. Health Perspect..

[15] Cordasco E.M., Demeter S.L., Kerkay J., van Ordstrand H.S., Lucas E.V., Chen T., Golish J.A. (1980). Pulmonary manifestations of vinyl and polyvinyl chloride (interstitial lung disease). Chest.

[16] Lilis R. (1980). Vinyl chloride and polyvinyl chloride exposure and occupational lung disease. Chest.

[17] Nielsen J., Fåhraeus C., Bensryd I., Akesson B., Welinder H., Lindén K., Skerfving S. (1989). Small airways function in workers processing polyvinylchloride. Int. Arch. Occup. Environ. Health.

[18] Hulin M., Simoni M., Viegi G., Annesi-Maesano I. (2012). Respiratory health and indoor air pollutants based on quantitative exposure assessments. Eur. Respir. J..

[19] Blanc P.D., Toren K. (1999). How much adult asthma can be attributed to occupational factors?. Am. J. Med..

[20] Oie L., Hersoug L.G., Madsen J.O. (1997). Residential exposure to plasticizers and its possible role in the pathogenesis of asthma. Environ. Health Perspect..

[21] Larsen S.T., Hansen J.S., Hammer M., Alarie Y., Nielsen G.D. (2004). Effects of mono-2-ethylhexyl phthalate on the respiratory tract in BALB/c mice. Hum. Exp. Toxicol..

[22] Ernst P., De Guire L., Armstrong B., Thériault G. (1988). Obstructive and restrictive ventilatory impairment in polyvinylchloride fabrication workers. Am. J. Ind. Med..

[23] Silva M.J., Slakman A.R., Reidy J.A., Preau J.L., Herbert A.R., Samandar E., Needham L.L., Calafat A.M. (2004). Analysis of human urine for fifteen phthalate metabolites using automated solid-phase extraction. J. Chromatogr. B Analyt. Technol. Biomed. Life Sci..

[24] Miller M.R., Hankinson J., Brusasco V., Burgos F., Casaburi R., Coates A., Crapo R., Enright P., van der Grinten C.P., Gustafsson P. (2005). ATS/ERS task force. Standardisation of spirometry. Eur. Respir. J..

[25] Global Initiative for Chronic Obstructive Lung Disease Global Strategy for the Diagnosis, Management and Prevention of Chronic Obstructive Pulmonary Disease. http://www.goldcopd.org.

[26] Gaudin R., Marsan P., Ndaw S., Robert A., Ducos P. (2011). Biological monitoring of exposure to di(2-ethylhexyl) phthalate in six French factories: A field study. Int. Arch. Occup. Environ. Health.

[27] Fromme H., Bolte G., Koch H.M., Angerer J., Boehmer S., Drexler H., Mayer R., Liebl B. (2007). Occurrence and daily variation of phthalate metabolites in the urine of an adult population. Int. J. Hyg. Environ. Health.

[28] Koch H.M., Calafat A.M. (2009). Human body burdens of chemicals used in plastic manufacture. Philos. Trans. R. Soc. Lond. B Biol. Sci..

[29] Koch H.M., Rossbach B., Drexler H., Angerer J. (2003). Internal exposure of the general population to DEHP and other phthalates-determination of secondary and primary phthalate monoester metabolites in urine. Environ. Res..

[30] Wittassek M., Heger W., Koch H.M., Becker K., Angerer J., Kolossa-Gehring M. (2007). Daily intake of di(2-ethylhexyl)phthalate (DEHP) by German children—A comparison of two estimation models based on urinary DEHP metabolite levels. Int. J. Hyg. Environ. Health.

[31] Saravanabhavan G., Guay M., Langlois É., Giroux S., Murray J., Haines D. (2013). Biomonitoring of phthalate metabolites in the Canadian population through the Canadian health measures survey (2007–2009). Int. J. Hyg. Environ. Health.

[32] Frederiksen H., Nielsen J.K., Mørck T.A., Hansen P.W., Jensen J.F., Nielsen O., Andersson A.M., Knudsen L.E. (2013). Urinary excretion of phthalate metabolites, phenols and parabens in rural and urban Danish mother-child pairs. Int. J. Hyg. Environ. Health.

[33] Saravanabhavan G., Murray J. (2012). Human biological monitoring of diisononyl phthalate and diisodecyl phthalate: A review. J. Environ. Public Health.

[34] Calafat A.M., Wong L.Y., Silva M.J., Samandar E., Preau J.L., Jia L.T., Needham L.L. (2011). Selecting adequate exposure biomarkers of diisononyl and diisodecyl phthalates: data from the 2005–2006 National Health and Nutrition Examination Survey. Environ. Health Perspect..

[35] Park H.Y., Kim J.H., Lim Y.H., Bae S., Hong Y.C. (2013). Influence of genetic polymorphisms on the association between phthalate exposure and pulmonary function in the elderly. Environmental Res..

[36] Grün F., Blumberg B. (2006). Environmental obesogens: Organotins and endocrine disruption via nuclear receptor signaling. Endocrinology.

[37] Stahlhut R.W., van Wijngaarden E., Dye T.D., Cook S., Swan S.H. (2007). Concentrations of urinary phthalate metabolites are associated with increased waist circumference and insulin resistance in adult U.S. males. Environ. Health Perspect..

[38] Hatch E.E., Nelson J.W., Qureshi M.M., Weinberg J., Moore L.L., Singer M., Webster T.F. (2008). Association of urinary phthalate metabolite concentrations with body mass index and waist circumference: A cross-sectional study of NHANES data, 1999–2002. Environ. Health.

[39] Hsu J.F., Peng L.W., Li Y.J., Lin L.C., Liao P.C. (2011). Identification of di-isononyl phthalate metabolites for exposure marker discovery using *in vitro*/*in vivo* metabolism and signal mining strategy with LC-MS data. Anal. Chem..

